# Development of a Predictive Model of Tuberculosis Transmission among Household Contacts

**DOI:** 10.1155/2019/5214124

**Published:** 2019-07-30

**Authors:** Saibin Wang

**Affiliations:** Department of Respiratory Medicine, Jinhua Municipal Central Hospital, No. 365, East Renmin Road, Jinhua 321000, Zhejiang Province, China

## Abstract

**Background:**

Household contacts of patients with tuberculosis (TB) are at great risk of TB infection. The aim of this study was to develop a predictive model of TB transmission among household contacts.

**Method:**

This was a secondary analysis of data from a prospective cohort study, in which a total of 700 TB patients and 3417 household contacts were enrolled between 2010 and 2013 at two study sites in Peru. The incidence of secondary TB cases among household contacts of index cases was recorded. The LASSO regression method was used to reduce the data dimension and to filter variables. Multivariate logistic regression analysis was applied to develop the predictive model, and internal validation was performed. A nomogram was constructed to display the model, and the AUC was calculated. The calibration curve and decision curve analysis (DCA) were also evaluated.

**Results:**

The incidence of TB disease among the contacts of index cases was 4.4% (149/3417). Ten variables (gender, age, TB history, diabetes, HIV, index patient's drug resistance, socioeconomic status, spoligotypes, and the index-contact share sleeping room status) filtered through the LASSO regression technique were finally included in the predictive model. The model showed good discriminatory ability, with an AUC value of 0.761 (95% CI, 0.723–0.800) for the derivation and 0.759 (95% CI, 0.717–0.796) for the internal validation. The predictive model showed good calibration, and the DCA demonstrated that the model was clinically useful.

**Conclusion:**

A predictive model was developed that incorporates characteristics of both the index patients and the contacts, which may be of great value for the individualized prediction of TB transmission among household contacts.

## 1. Introduction

Tuberculosis (TB) continues to be a heavy global burden. It is estimated that 10 million persons worldwide were newly infected in 2017, including 5.8 million men, 3.2 million women, and 1 million children (≤15 years) [1]. TB is the leading cause of death caused by a single pathogen infection, and its mortality rate in 2017 reached 16% [1]. Early diagnosis of TB is very important [2]. However, the occurrence of TB infection is generally difficult to predict, and delays in diagnosis are common.

As an infectious pathogen, *Mycobacterium tuberculosis* is characterized by its ability to be transmitted and to cause disease in another host. Individuals in contact with active TB patients are susceptible to TB, and household contacts are considered to be at higher risk due to their constant exposure to infected patients [3]. Several studies have revealed that a number of clinical, environmental, and socioeconomic variables (such as human immunodeficiency virus (HIV) positive, diabetic, and poverty status) may affect the incidence of TB in contacts [3]. In addition, previous studies involving both animal models and human patients have demonstrated that the pathogenicity of drug-resistant and drug-susceptible *Mycobacterium tuberculosis* differs [3–5]. Previously, although several predictive models of TB infection based on nosocomial small samples have been established and showed certain application value in predicting the duration of TB patient isolation [6–8], to the best of our knowledge, there is currently no model available for prediction of TB transmission in communities or households.

In the present study, based on a completed 3-year prospective cohort study [3], the clinical, environmental, and socioeconomic characteristics of both index TB patients and their household contacts were retrospectively investigated to develop a predictive model of TB transmission.

## 2. Methods

### 2.1. Study Population and Ethics

This study was based on a previous prospective cohort study conducted in Peru [3], which is classified by the WHO as a high TB burden country [1]. A total of 700 TB patients (213 multidrug-resistant tuberculosis (MDRTB) cases and 487 drug-susceptible cases) and 3417 household contacts were enrolled between September 2010 and September 2013 for the study. In the previously published study [3], Grandjean et al. have clearly stated that the ethical approval of this study was obtained from the Institutional Review Board of Universidad Peruana Cayetano Heredia (IRB00001014), and informed written consent was obtained from all participants.

### 2.2. Variable Collection

For this study, the following variables were collected from both TB patients and their household contacts: gender, age, previous TB history (yes or no), HIV infection status (yes or no), coexisting diabetes (yes or no), socioeconomic status (divided into three levels based on the scoring system used in the Peruvian National Census) [3], employment status (unemployed, working, student, or unknown), and secondary education status. In addition, the following variables were collected from TB patients: drug resistance status (MDRTB indicated resistant to at least rifampicin and isoniazid, and drug-susceptible indicated susceptible to both rifampicin and isoniazid), alcohol and tobacco use, spoligotypes (stratified based on the SpolDB4 database), sputum smear grade, mean cough duration, hospitalization history, and side effects of treatment. Household contacts were defined as persons living in the same room with TB patients for more than one day a week. A household contact TB infection was defined as the development of TB disease occurring after the diagnosis of TB in the index patient [3]. TB was diagnosed based on positive sputum smears or cultures, chest X-rays, or a clinical diagnosis that resulted in initiation of antituberculosis treatment [3]. In the case of household contacts, information as to whether the person slept in the same room with the TB patient and the time of occurrence of TB disease was also collected.

### 2.3. Statistical Analysis

The multiple imputation method was used for dealing with missing values. The baseline characteristics of the study population were summarized as the number and the percentage. In this study, we followed the methods of Wang 2019 [9]. The least absolute shrinkage and selection operator (LASSO) regression method was used for data dimension and variable selection. Multivariate logistic regression analysis with backward stepwise selection using the likelihood ratio test with Akaike's information criterion [10] was applied to develop a predictive model of TB infection in household contacts. A nomogram was constructed to present the model. The discriminatory capacity of the model was determined by calculating the area under the curve (AUC). Internal validation by means of the bootstrap method (resampling = 500) was performed [11]. A calibration curve was plotted to evaluate the model together with the Hosmer–Lemeshow test, and decision curve analysis (DCA) was performed to assess the clinical usefulness of the model [12]. Statistical analysis was conducted with *R* software (version 3.5.1). A *P* value of <0.05 was considered statistically significant.

## 3. Results

In this study cohort, 4.4% (149/3417) (95% confidence interval (CI), 3.7–5.1%) of household contacts developed TB disease. The median (25%–75% interquartile) time for the first TB infection of household contacts was 153 (52–264) days. Demographic data of TB patients and household contacts are shown in Tables 1 and 2, respectively.

**Figure 1:**
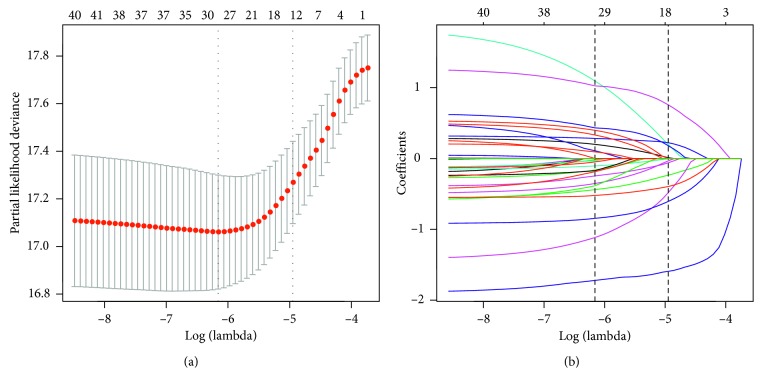
Selection of predictors using the LASSO regression analysis with 10-fold cross-validation. (a) Tuning parameter (lambda) selection of deviance in the LASSO regression based on the minimum criteria (left dotted line) and the 1-SE criteria (right dotted line). (b) A coefficient profile plot was produced against the log (lambda) sequence. In the present study, predictor's selection was according to the 1-SE criteria (right dotted line), where 11 nonzero coefficients were selected. SE, standard error.

Of 22 variables collected from the study cohort, 11 variables were selected based on nonzero coefficients calculated by the LASSO regression analysis (Figure 1). These variables were contact's gender, age, previous history of TB, diabetes, HIV infection status, index TB patient's drug resistance status, diabetes, socioeconomic status, educational status, spoligotypes, and whether the index case and the contact slept in the same room.

Multivariate logistic regression analysis was conducted including the aforementioned 11 variables selected by the LASSO regression analysis. Backward stepwise selection was applied to develop a predictive model by using the likelihood ratio test with Akaike's information criterion. Ten variables (all the variables described above, with the exception of educational status) were eventually incorporated into the model.

**Figure 2:**
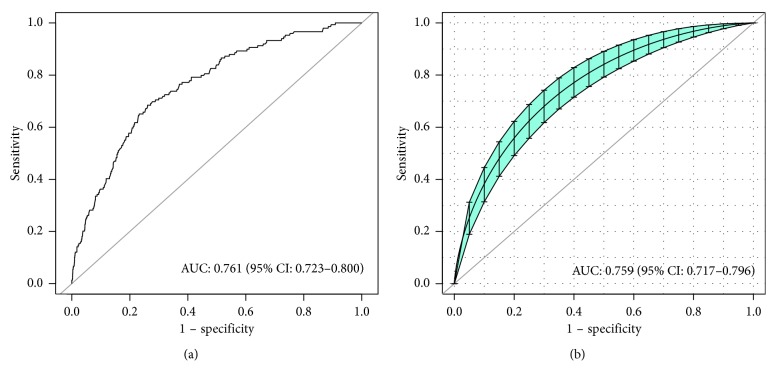
The receiver operating characteristic of the model and in the internal validation cohort. (a) AUC of the predictive model (representative the discriminatory ability of the model) and (b) AUC of the internal validation (bootstrap resampling = 500). The dotted vertical lines represent 95% confidence interval. AUC, area under the curve.

**Figure 3:**
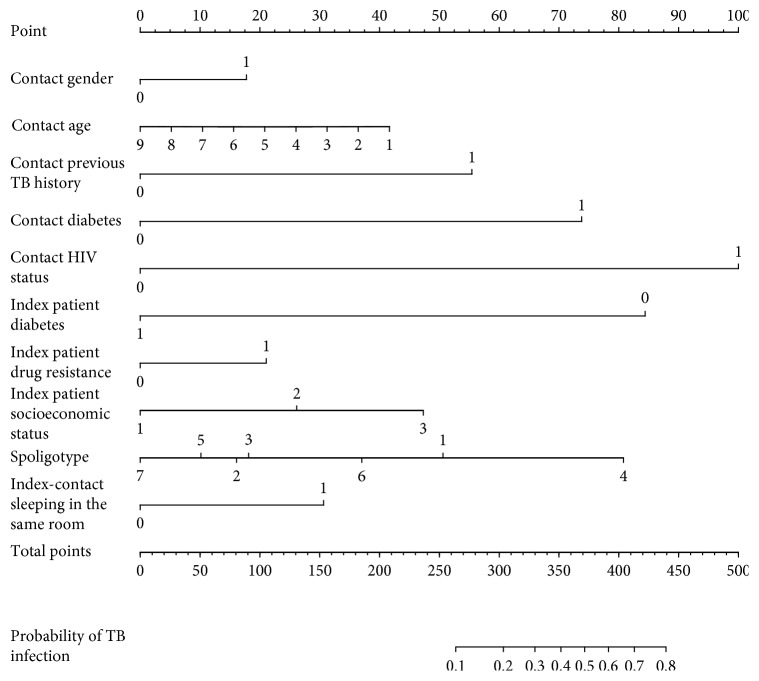
Nomogram for TB transmission in households exposed to TB patients and its algorithm. First, find point for each variable of a contact on the uppermost rule; then add all scores together and find the total point on the “Total points” rule. At last, the corresponding predicted probability of TB could be found on the lowest rule. Codes annotation: contact gender: 0, female; 1, male. Contact age (years): 1, 0 < and ≤ 10; 2 : 10 < and ≤ 20; 3, 20 < and ≤ 30; 4, 30 < and ≤ 40; 5, 40 < and ≤ 50; 6, 50 < and ≤ 60; 7, 60 < and ≤ 70; 8, age > 70; 9, unknown. Contact previous TB history: 0, no; 1, yes. Contact diabetes: 0, no; 1, yes. Contact HIV status: 0, no; 1, yes. Index patient diabetes: 0, no; 1, yes. Index patient drug resistance: 0, no; 1, yes. Index patient socioeconomic status (based on a scoring system used in the Peruvian National Census) [3]: 1, lower tertile; 2, middle tertile; 3, upper tertile. Spoligotype: 1, Haarlem; 2, Beijing; 3, Latin American Mediterranean; 4, *T* strain; 5, other Euro-American strains; 6, orphan or no family; 7, unknown. Index-contact sleeping in the same room: 0, no; 1, yes.

As shown in Figure 2, the AUC for the predictive model was 0.761 (95% CI, 0.723–0.800), while the AUC for the internal validation using the bootstrap method (resampling = 500) was 0.759 (95% CI, 0.717–0.796). A nomogram was also constructed based on the predictive model (Figure 3), providing a quantitative tool to predict the probability of TB transmission in household contacts.

**Figure 4:**
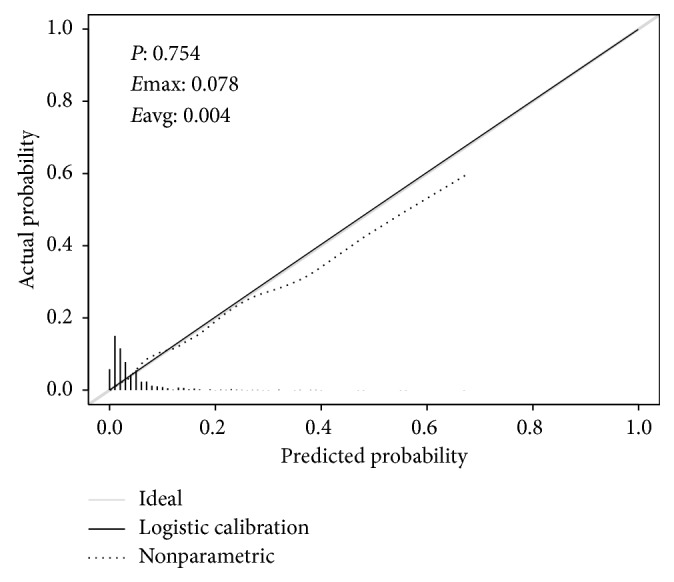
Calibration curves of the predictive model. It shows the degree of consistency between the predicted risks of TB transmission in households exposed to TB patients and observed outcomes. The shadow line represents a perfect prediction by an ideal model, and the dotted line shows the performance of the model. The Hosmer–Lemeshow test yielded a *P* value of 0.754, Emax of 0.078, and Eavg of 0.004. *E*, difference in predicted and calibrated probabilities between calibration and area under the curve.

A good calibration is shown in Figure 4. The Hosmer–Lemeshow test yielded nonsignificant statistical value (*P*=0.754), with an Emax of 0.078 and Eavg of 0.004, suggesting that there was no departure from a perfect fit between prediction and observation.

**Figure 5:**
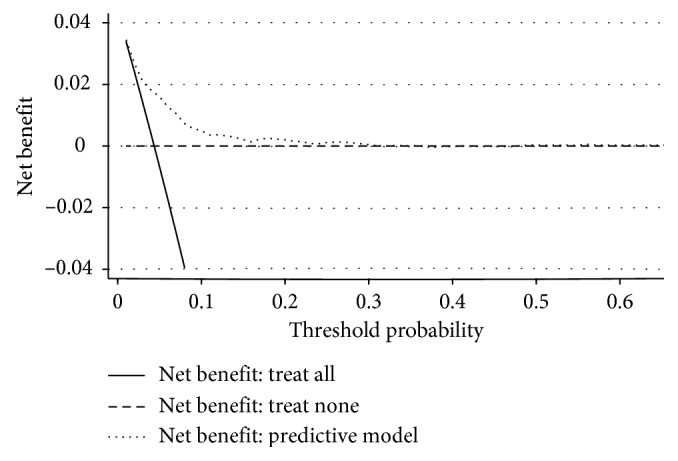
Decision curve analysis of the predictive model. The dotted line represents the model. Area among the dotted line, the solid line, and the dashed line represents the net benefit from applying this model for prediction of TB transmission in households exposed to TB patients. The decision curve shows that when the threshold probability of a patient is <30%, application of this mode adds more benefit than either the treat-all or the treat-none strategies.

The DCA for the model is presented in Figure 5. The decision curve showed that when the threshold probability of TB transmission in household contacts was <30% based on the predictive model, application of this model to predict household contact TB infection would add more benefit than either the treat-all or treat-none strategies.

## 4. Discussion

In the current study, a predictive model of transmission risk among household contacts exposed to index TB cases was developed. This model incorporates 10 predictors: contact's gender, age, previous TB history, diabetes, HIV infection status, index patient diabetes, index TB patient's drug resistance status, socioeconomic status, spoligotypes, and the index-contact share sleeping room status. The model showed good discriminatory ability, with an AUC of 0761. Internal validation based on the bootstrap method (resampling = 500) yielded an AUC of 0.759. Moreover, the model showed good calibration and clinical usefulness.

TB transmission has always been a global health concern. Recently, the Global Tuberculosis Report 2018 released by the WHO indicated that the fight against TB is still ongoing [1]. The incidence of TB remains high, with 10 million new cases in 2017 [1]. Contact with active TB patients is the main mode of TB transmission, and due to their frequent exposure, household contacts may be at higher risk of TB infection than nonhousehold contacts [3].

In previous studies, several variables affecting TB transmission have been described. In both retrospective and prospective studies, it has been demonstrated that the incidence of TB in household contacts is higher in the case of drug-susceptible TB cases than in MDRTB index cases [3, 13]. Coexisting HIV infection or diabetes in household contacts has been confirmed to be a risk factor for TB infection [1]. In addition, a lower socioeconomic status is associated with a higher TB incidence [1, 3]. The *Mycobacterium tuberculosis* spoligotype signature can also influence the level of transmission in household contacts and in the community [3, 14]. As expected, an independent risk factor for TB transmission is the fact that contacts and TB patients share the same sleeping room [3]. However, the current high incidence of TB transmission is mainly attributed to unrecognized active TB cases; therefore, accurate identification of active index TB cases and prediction of the risk of TB infection are essential to prevent transmission [8]. To our knowledge, few studies have described TB transmission risk prediction models. On the other hand, delays in TB diagnosis commonly occur in clinical practice. Atypical clinical presentations and clinician inexperience are partly responsible for delayed TB diagnosis [15, 16]. In this respect, predictive models can play an important role, helping clinicians or healthcare providers predict the probability of TB infection and guiding their clinical decision making to achieve a timely diagnosis.

Based on the retrospective analysis of a small sample, in 1997, Mytotte et al. [16] described a predictive model for evaluating the risk of TB infection among patients in isolation in a New York hospital. The model included four predictors: a positive acid-fast sputum smear, localized chest radiographic findings, residence in a correctional facility, and history of weight loss. These authors concluded that application of the model was partly responsible for a decrease in the mean duration of patient isolation. Noteworthy, approximately 40% of cases in their derivation cohort resided in correctional facilities, and almost 60% were HIV positive. A decade later, another study by Rakoczy et al. [6] with a smaller sample size (a total of 81 in the derivation and validation cohorts) derived and validated a clinical prediction score for patients with suspected TB, also using four predictors: chronic symptoms, upper lobe disease on chest radiograph, foreign-born status, and immunocompromised state other than HIV infection. These authors pointed out that the model could improve compliance with airborne precautions. In addition, EI-Solh et al. [14] developed an artificial neural network for predicting active pulmonary TB using clinical and radiographic variables and based on a nonconcurrent prospective study with 563 isolation episodes in the derivation and 119 in the validation. They reported that the artificial neural network could identify patients with active pulmonary TB more accurately than physicians' clinical assessments. Of note, the above three models were based on nosocomial populations and were not developed to predict TB transmission between index cases and contacts. Therefore, these models are not suitable to predict TB transmission in communities or households.

The model in the present study was developed based on a 3-year prospective study, which included 700 index TB patients and 3417 household contacts [3]. Clinical, demographic, and socioeconomic variables were investigated in both index patients and contacts. Candidate predictors included in the model were filtered by LASSO regression analysis, which is considered superior to selecting predictors by univariate analysis [17]. All these 10 predictors are easily available clinically. A predictive model would be considered to have applied potential only when the discriminatory capacity and calibration, as well as DCA of the model, are good in performance [9, 18]. The predictive model showed good discriminatory ability (AUC: 0.761) and calibration. Moreover, the DCA evaluation showed its clinical usefulness. Specifically, it demonstrated that utilization of the predictive model would be more beneficial than either the treat-all or treat-none strategies. In addition, we also constructed a nomogram to facilitate the application of the model.

Some limitations of this predictive model are worth noting. First, prediction of TB transmission needs to take into account regional differences in TB epidemiology. This model was based on a 3-year prospective study conducted in southern Lima and Callao, Peru [3]. Therefore, determining whether this predictive model is applicable to other regions requires further verification. Second, there was a time span between latent infection and TB diagnosis. Thus, in a small number of household contacts diagnosed with TB within a very short time frame, it cannot be determined whether the contact was infected following exposure or if there was a preexisting latent infection. Third, some potentially relevant clinical variables, such as other complications (except for diabetes and HIV infection), the regimens and duration of treatment of the index patient, and radiological extension of disease were not included in the analysis because they were not available in the original data [19].

Despite these limitations, this study is the first to develop a predictive model for transmission among household contacts of TB patients.

## 5. Conclusions

A TB transmission risk prediction model for household contacts was developed, which incorporates characteristics of both TB patients and contacts. This model showed good discriminatory ability and may be of great value to facilitate the prediction and management of TB transmission in households.

## Figures and Tables

**Table 1:** Demographic data of index TB patients.

Characteristic	Index patients (*n* = 700)
Mean age, (years)	33
Male, *n* (%)	273 (39.0)
Alcohol use (≥one unit/day), *n* (%)	79 (11.3)
Tobacco use (any cigarettes/week), *n* (%)	108 (15.4)
Previous TB history, *n* (%)	130 (18.6)
HIV positive, *n* (%)	38 (5.4)
Coexisting diabetes, *n* (%)	40 (5.7)
Socioeconomic status^*∗*^, *n* (%)	
1	288 (41.1)
2	210 (30.0)
3	202 (28.9)
Completed secondary education, *n* (%)	417 (59.6)
Employment status, *n* (%)	
Unemployed	378 (54.0)
Working	235 (33.6)
Student	84 (12.0)
Unknown	3 (0.4)
Spoligotype family (SpolDB4 database), *n* (%)	
Haarlem	143 (20.4)
Beijing	72 (10.3)
Latin American Mediterranean	92 (13.2)
*T*	143 (20.4)
Other Euro-American^*∗∗*^	61 (8.7)
Orphan/no family	75 (10.7)
Unknown (no data)	114 (16.3)
Mean cough duration (weeks)	6.3
History of hospitalization, *n* (%)	89 (12.7)
Any side effects of treatment, *n* (%)	351 (50.1)
Sputum smear grade, *n* (%)	
0	67 (9.6)
1	197 (28.1)
2	180 (25.7)
3	234 (33.4)
Unknown	22 (3.2)
MDRTB patient, *n* (%)	213 (30.4)

^*∗*^Divided into three levels based on the scoring system used in the Peruvian National Census; ^*∗∗*^“Other Euro-American” includes strains from the S family, the X family, and strains that were present in the SpolDB4 database but had not been assigned a family yet [3]. TB, tuberculosis; HIV, human immunodeficiency virus; MDRTB, multidrug-resistant tuberculosis.

**Table 2:** Demographic data of household contacts.

Characteristic	Household contacts (*n* = 3417)
Age stratum (years), *n* (%)	
0–10	500 (14.6)
10–20	625 (18.3)
20–30	602 (17.6)
30–40	673 (19.7)
40–50	385 (11.3)
50–60	329 (9.6)
60–70	174 (5.1)
>70	118 (3.5)
Unknown	11 (0.2)
Male, *n* (%)	1698 (49.7)
Previous TB history, *n* (%)	583 (17.1)
HIV positive, *n* (%)	20 (0.6)
Coexisting diabetes, *n* (%)	41 (1.2)
Completed secondary education, *n* (%)	1540 (45.1)
Employment status, *n* (%)	
Unemployed	825 (24.1)
Working	1348 (39.4)
Student	883 (25.9)
Unknown	361 (10.6)
Index-contact case sleeping in the same room, *n* (%)	640 (18.7)
Second cases of TB, *n* (%)	149 (4.4)

TB, tuberculosis; HIV, human immunodeficiency virus.

## Data Availability

The data used in this study can be downloaded from the Dryad database (http://www.datadryad.org).
